# Evolutionary model for the unequal segregation of high copy plasmids

**DOI:** 10.1371/journal.pcbi.1006724

**Published:** 2019-03-05

**Authors:** Karin Münch, Richard Münch, Rebekka Biedendieck, Dieter Jahn, Johannes Müller

**Affiliations:** 1 Institute of Microbiology, Technische Universität Braunschweig, Spielmannstr. 7, D-38106 Braunschweig, Germany; 2 Braunschweig Integrated Centre of Systems Biology (BRICS), Technische Universität Braunschweig, D-38106 Braunschweig, Germany; 3 Centre for Mathematical Sciences, Technische Universität München, Boltzmannstr. 3, D-85747 Garching/Munich, Germany; 4 Institute of Computational Biology, Helmholtz Zentrum München - German Research Center for Environmental Health, Ingolstädter Landstr. 1, D-85764 Neuherberg, Germany; Duke University, UNITED STATES

## Abstract

Plasmids are extrachromosomal DNA elements of microorganisms encoding beneficial genetic information. They were thought to be equally distributed to daughter cells during cell division. Here we use mathematical modeling to investigate the evolutionary stability of plasmid segregation for high-copy plasmids—plasmids that are present in up to several hundred copies per cell—carrying antibiotic resistance genes. Evolutionary stable strategies (ESS) are determined by numerical analysis of a plasmid-load structured population model. The theory predicts that the evolutionary stable segregation strategy of a cell depends on the plasmid copy number: For low and medium plasmid load, both daughters receive in average an equal share of plasmids, while in case of high plasmid load, one daughter obtains distinctively and systematically more plasmids. These findings are in good agreement with recent experimental results. We discuss the interpretation and practical consequences.

## Author summary

In the last years, it becomes more and more clear that heterogeneity in isogenic bacterial populations is rather the rule than the exception. This observation is interesting as it reveals the complex social life of bacteria, and also because of tremendous practical implications in medicine, biotechnology, and ecology. The central questions in this field are the identification of the underlying proximate causes (molecular mechanisms) on the one hand and on the other hand the identification of ultimate causes (evolutionary forces) that shape the social life of bacteria. We focus on plasmid dynamics, in particular on plasmid segregation. Recent experiments showed that plasmid segregation depends on the plasmid load. We identify possible evolutionary factors that shaped this process. It turns out that the ambivalence in the effect of plasmids—advantageous if present in low copy numbers, a metabolic burden if present in high copy numbers—is able to explain the experimental observations. The experimental findings can be interpreted as a variant of the principle of division of labor, as it is well known from e.g. persister cells or sporulation. Our model extends the theory of unequal segregation of damage to the ambivalent role of plasmids. Similarly, it is known that certain gene regulatory proteins are acting in a dose-dependent manner. Due to differences in their cellular concentrations and in their affinities to various target promoters, differential gene expression patterns are achieved. Consequently, tight concentration control is observed [1]. Another example is the strict copy-dependent utilization of autolysins during cell division. These enzymes carefully open the bacterial cell wall to allow for its extension [2]. Overproduction of these enzymes leads to cell lysis [3]. These molecules are in principle also candidates for a segregation characteristic similar to that of high copy plasmids described here.

## Introduction

Plasmids are circular or linear pieces of DNA of different size (several 1000 to 100,000 bp) encoding beneficial genetic information for their microbial hosts replicated independently from the chromosome(s) [4]. In some cases, plasmids are essential for the growth and survival of the microorganisms under certain environmental conditions [5]. The copy number of plasmids per cell is dependent on the nature of the origin of replication (ori) and varies between 1 to 2 copies for low copy plasmids to 50 to 800 for high copy plasmids [6]. Often plasmids possess genes for enzymes or transporters mediating resistance to antibiotics [7], sporulation, or germination [8]. In the present paper, we focus on high-copy plasmids. In contrast to low-copy plasmids that often incorporate a distinct active mechanism of segregation ensuring that during cell division each daughter inherits at least one copy [9–11], high-copy plasmids are mainly segregated by random diffusion [12–14]. Only a few years ago equal distribution of plasmids between daughter cells during cell division was assumed. Now we know that at least some high-copy plasmids are distributed unequally between their production hosts [15]. The plasmids investigated in those experiments are of particular interest for biotechnological use. Unequal plasmid segregation may lead to the commercially important factor of cost-intensive feeding of non-productive microbial cells. Therefore, experimental and theoretical approaches are required to elucidate the underlying evolutionary and molecular mechanisms as a foundation for their directed improvement.

In detail, recent experimental findings indicate that the segregation of high copy number plasmids depends on the copy number within the mother cell [11, 15]. If the copy number is in a low or in a medium range, both daughters receive in average the same amount of plasmids. If the copy number of the mother is high, one daughter cell receives systematically more plasmids that the other. The consequences of unequal plasmid segregation have been addressed before [16, 17], but did not receive much attention; in these papers, evolutionary theory has not been taken into account. The aim of the present paper is exactly to focus on evolutionary forces in order to shed some light on segregation. In particular, we aim to identify evolutionary mechanisms that explain the experimental findings.

There are several models for plasmid dynamics introduced in the literature, see e.g. [18–25]. The present model takes up ideas particularly developed in [18] and [24], where a bacterial population structured by copy number is described. We use, as [24], a copy-number dependent plasmid reproduction rate, and extend the model (in a similar spirit as e.g. [26]) by possibly unequal segregation and bacterial reproduction, both depending on the plasmid copy-number. In the supplementary information (S1 Text, effect of horizontal plasmid transmission) we furthermore investigate the effect of horizontal plasmid transfer. Moreover, in the natural environment of bacteria, antibiotics are present only from time to time. Most likely, evolutionary forces act in the setting of a fluctuating environment. An observation in recent years is the importance of fluctuating environment on phenotypic heterogeneity [27–32]. We extend our model by fluctuating environment (see section “Long-term behavior” below).

Based on that model, we use ideas of Adaptive Dynamics [33, 34] to identify evolutionary stable segregation strategies (ESSS). According to Fisher’s fundamental theorem, in the situation described above, the evolutionary stable trait maximizes the average fitness of the population, that is, the average growth rate of the bacterial community [35]. We numerically compute the average population growth rate for a given segregation strategy, and find, again by means of numeric analysis, the ESSS. It turns out that for a wide parameter range, the ESSS resembles qualitatively the observed segregation pattern. In order to understand this finding intuitively, we come up with a simplified model that is close to Chao’s theory for damage segregation [26].

## Results

### Construction of the mathematical model

In order to develop a new mathematical model (incorporating unequal plasmid segregation) of a bacterial population carrying a high-copy plasmid, we consider a well-mixed, homogeneous recombinant bacterial population, structured by copy number of the corresponding recombinant plasmid (find the details of the model below, after the discussion). The plasmids we have in mind protect their carrier against certain antibiotics, but result in specific metabolic costs [36]. In the natural environment, local antibiotic concentrations are usually low but functional. Under these conditions protection does not depend on the plasmid copy number—in principle, if one plasmid is present, a cell is protected—nevertheless, the metabolic burden increases with the copy number.

We model the system on two different levels: (1) plasmid reproduction within bacteria, and (2) bacterial reproduction/division. With respect to the first point, it is well known that plasmid reproduction is tightly controlled [37–40]. The plasmid production rate is well approximated by logistic growth. That is, if we find *z* plasmids within a cell, the plasmid reproduction rate is given by $b\;z\;(1-z/\hat{z})$. In the main part of the paper, we neglect horizontal plasmid transfer [41, 42] but show in the SI (S1 Text, effect of horizontal plasmid transmission) that our results are stable w.r.t. this mechanism.

**Fig 1:**
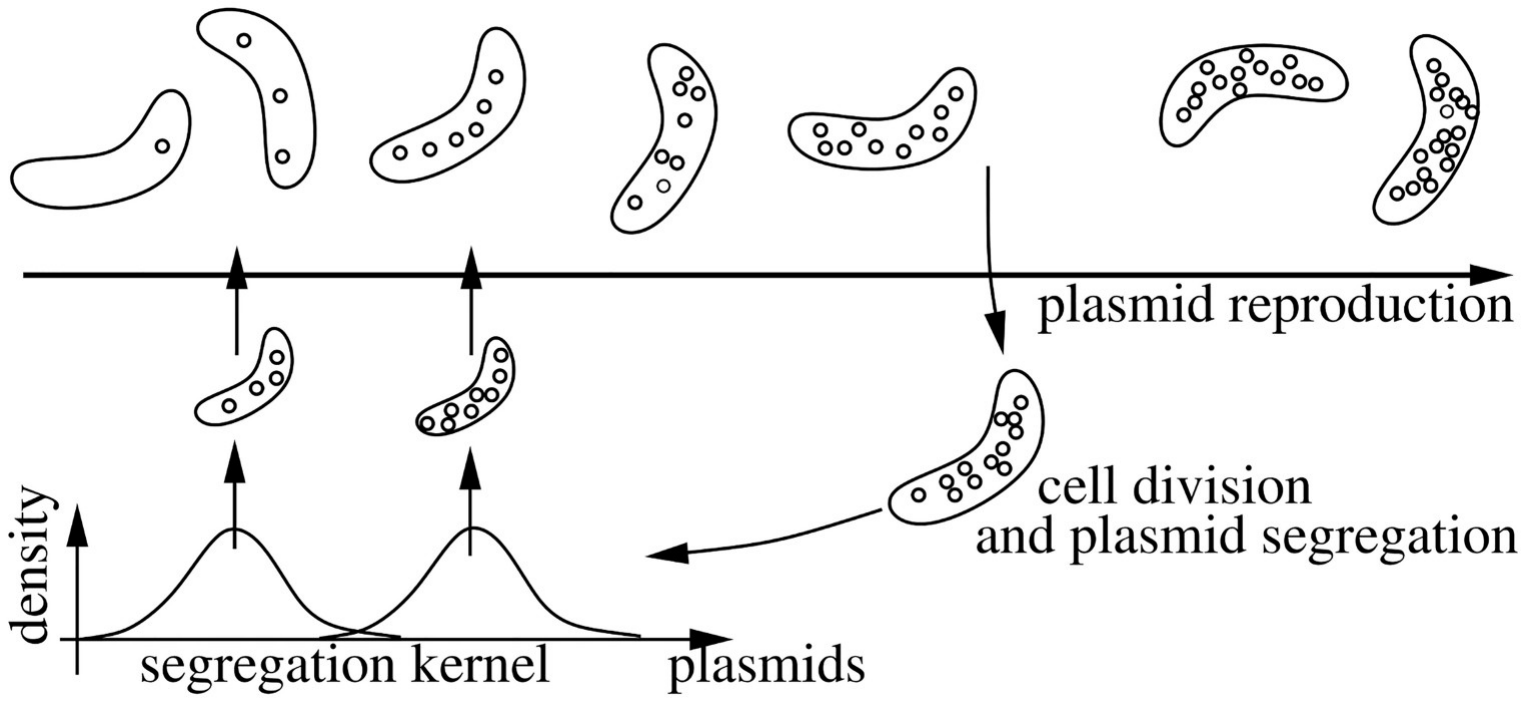
Plasmid and population dynamics: Plasmid reproduction increases the copy number within a cell, cell division dilutes the plasmids and reduces the copy number per cell. The segregation distribution depends on the number of plasmids in the mother cell.

To come to the second point, we take the effect of the metabolic burden induced by plasmids on the cell cycle into account. We assume that cells containing *z* plasmids divide at rate $\beta_{0}\left(1-z/\hat{z}\right)$ (see section “parameters and results of the sensitivity analysis” below for a discussion of the rationale behind this choice). If cells divide, the plasmids are distributed to the daughter cells. Plasmid segregation is modeled by a stochastic process. If a cell containing *z* plasmids divides, we assume that the copy number inherited by one daughter is described by a binomial distribution with *N* = *z* and some probability *p*_*z*_. The other daughter receives the remaining plasmids (Fig 1). If *p*_*z*_ = 1/2, segregation is equal in both daughters and both receive in average the same amount of plasmids. If *p*_*z*_ distinctively differs from 1/2, one daughter systematically receives more plasmids. As *p*_*z*_ depends on the number of plasmids *z* in the mother cell, the model allows for a plasmid-load dependent segregation characteristic.

The asymptotic copy number distribution is shaped by two counter-acting mechanisms. The plasmid replication within cells (assumed to follow a logistic dynamics with a carrying capacity/maximal copy number $\hat{z}$) increases the plasmid load, while cell division decreases the copy number by distributing the plasmids on two daughter cells (Fig 1).

### Long-term behavior and definition of evolutionarily stable strategies

According to Fisher’s fundamental theorem about selection, evolutionary forces maximize the average fitness of the population [35]. In the present simple setting, the average fitness of the population is given by the long-term population growth. A segregation characteristic maximizing this long-term population growth forms an evolutionary stable strategy (ESS) [33, 34]. In mathematical terms, the long-term average growth rate is defined as the Lyapunov exponent of the model [43]. In a linear model with constant coefficients, the Lyapunov exponent agrees with the dominant eigenvalue [44]. Without horizontal plasmid transfer, the model is reducible. In contrast to irreducible models (which have a unique non-negative exponentially growing solution), we have two different non-negative exponentially growing solutions: One describes the population without plasmids, and one the solution of the population bearing plasmids. We compute the Lyapunov coefficient for the population that carries plasmids numerically and used the method of steepest ascent to numerically determine the segregation strategy that maximizes the Lyapunov exponent. In doing so, we solely focus on the plasmid-bearing subpopulation. The plasmid-free subpopulation, however, might have a higher Lyapunov exponent, that is, a higher fitness. In this case, the plasmid-bearing population is outcompeted by the plasmid-free subpopulation, and the plasmid gets lost. This is, in particular, the case if antibiotics are never present in the environment. If antibiotics appear—at least from time to time (switching environment, see section “Long-term behavior”)—and superimpose a fitness disadvantage to plasmid-free cells that is high enough, the plasmid carrying cells have an advantage. Though plasmids come with a certain burden (fitness disadvantage), the advantage due to the protection outweighs their disadvantages (see Theorem 1 below). The plasmids will persist in the population, and Adaptive Dynamics predicts that evolutionary forces drive the segregation strategy towards the ESSS.

### ESSS

**Fig 2:**
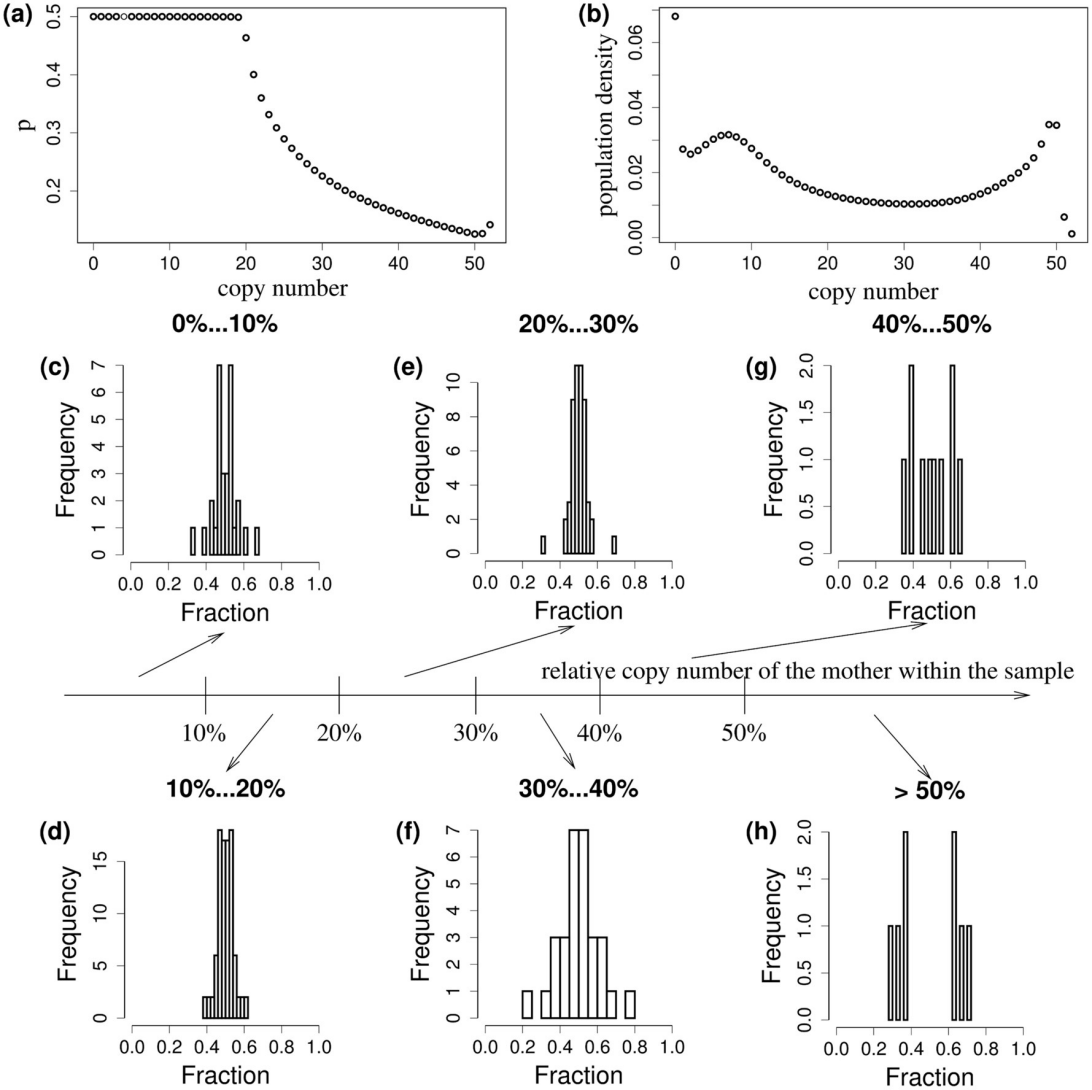
(a) ESSS for the parameters stated in Table 1 (see also the discussion of parameters in section “parameters and sensitivity analysis”). (b) Population density over copy number for this segregation strategy. (c)-(h) Experimental segregation characteristics: We stratify the mother cells according the number of plasmids (fluorescence). The histograms of the fraction of plasmids a daughter inherits is presented (if *f*_*i*_ are the fluorescence of the two daughter cells, we show the histogram of *f*_1_/(*f*_1_ + *f*_2_), *f*_2_/(*f*_1_ + *f*_2_). Note that we have thus always symmetry w.r.t. 1/2).

**Fig 3:**
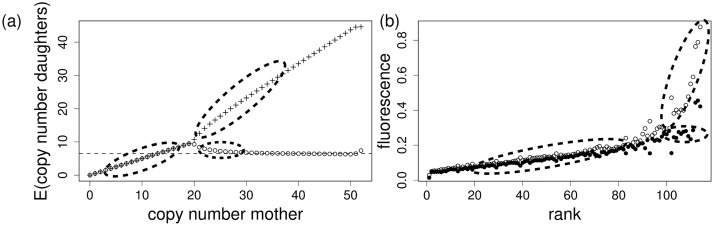
(a) Theory. Expected copy number for the two daughters over copy number of the mother (ESSS for the parameters stated in Table 1), see also the discussion of parameters in section “parameters and sensitivity analysis”. The dashed, horizontal line indicates the asymptotic copy number of the cell with fewer plasmids will receive if the mother’s copy number is very high. (b) Fluorescence data. Mother cells are ordered by increasing copy number (given by fluorescence, defining the rank of the mother). Fluorescence [AU] of the to daughters (open circle: higher fluorescence, bullets: smaller fluorescence) is drawn over the rank of the mother.

Numerical analysis revealed that the strategy maximizing the average growth rate varies with the copy number (Figs 2(a) and 3, left panel). For a small or medium plasmid copy number, *p*_*z*_ = 1/2 is optimal. This is due to the fact that cells without plasmids will most likely be killed in episodes with antibiotics. *p*_*z*_ = 1/2 maximizes the chance that both daughter cells receive at least some plasmids, and in this, the survival chance of both daughters is maximized.

However, the optimal segregation characteristic also avoids plasmid copy numbers that seriously decrease the bacterial reproduction due to the metabolic burden. Therefore, in the case of high copy numbers, the cells choose an unequal segregation mechanism. One daughter is pushed back to copy numbers that on the one hand ensure protection, also after several further cell divisions, and on the other hand are small enough not to induce a large metabolic burden. This strategy necessarily produces sister cells with a high copy number and a high metabolic burden. Those sister cells are basically lost for generating population growth. Fig 3 (left panel) shows that the expected copy number of one daughter in the ESSS is relatively constant (if the copy number of the mother exceeds 30). Accordingly, the copy number of the second daughter increases linearly with the mothers’ copy number and becomes rather high. The ESSS optimizes the copy number of one daughter cell only.

The resulting asymptotic copy number distribution is bimodal (Fig 2(b)). There is a large, active subpopulation with relatively few plasmids, while a second (smaller) subpopulation appears at the carrying capacity of plasmids within a cell. That subpopulation will be inactive due to the high metabolic burden. A third subpopulation is not shown in the figure, as we concentrate in the present section on the plasmid-bearing bacteria only: there also exists a subpopulation without plasmids, optimized for the antibiotic-free environment.

This finding is rather stable w.r.t. the choice of parameters (see below, section “Parameters and results of the sensitivity analysis”): Essentially, the plasmid growth rate *b* needs to be in a similar range or larger than the cell division rate *β*_0_, and the plasmids need to come with a heavy metabolic burden if present in a large number. Other parameters and mechanisms as the plasmid carrying capacity $\hat{z}$ or horizontal plasmid transfer only play a minor role.

### Comparison with experimental data

In former experiments, recombinant plasmids encoding resistance against the antibiotic tetracycline were further manipulated, such that they cause the production of recombinant green fluorescent protein GFP in the plasmid carrying cell (find details in [15]). It was shown by a quantitative polymerase chain reaction and corresponding fluorescence in situ hybridization that the amount of fluorescence of a cell caused by GFP correlates with the plasmid copy number per cell [15]. Using time-lapse microscopy, the offspring of one single cell has been followed for several generations. In this way, it was possible to obtain information about the plasmid copy number in the mother as well as in both daughter cells [15]. These data, in turn, carry information about the plasmid-dependent segregation probabilities (Fig 2). In order to extract copy-number-dependent segregation strategies, daughter cells are grouped according to the fluorescence of the mother (the lowest 10%, 10%-20%, etc., in relation to the observed maximal fluorescence). Then, the relative amounts of fluorescence of the daughters are computed

$$\begin{array}{c} \frac{\text{fluorescence}\;\text{of}\;\text{a}\;\text{daughter}}{\text{sum}\;\text{of}\;\text{fluorescence}\;\text{in}\;\text{both}\;\text{daughtes}}\approx \frac{\text{plasmids}\;\text{in}\;\text{a}\;\text{daughter}}{\text{plasmids}\;\text{in}\;\text{the}\;\text{mother}} \end{array}$$

and represented as a histogram. If *f*_1_ is the fluorescence for one daughter, and *f*_2_ for the other daughter, the histogram is based the fraction of fluorescence contained in both daughters, that is, on the data points *f*_1_/(*f*_1_ + *f*_2_) and *f*_2_/(*f*_1_ + *f*_2_). Since

$$\begin{array}{c} \frac{f_{1}}{f_{1}+f_{2}}=1-\frac{f_{2}}{f_{1}+f_{2}}\;\Rightarrow \;\frac{f_{1}}{f_{1}+f_{2}}-\frac{1}{2}=\frac{1}{2}-\frac{f_{2}}{f_{1}+f_{2}}, \end{array}$$

the histograms are perfectly symmetrical about 0.5. This histogram shows the segregation structure of the corresponding mother cells. We find that these histograms are concentrated at 1/2 for the lowest 40% (Fig 2(c) and 2(d)). This finding corresponds to a segregation probability equal or at least close to *p*_*z*_ = 1/2. Above 40%, the histograms show a bimodal distribution (Fig 2(g) and 2(h)), indicating that *p*_*z*_ is distinctively below 1/2. Another way to compare model and measurements is to address the average number of plasmids in the daughter cells in dependence on the mothers’ plasmid copy number (Fig 3, left panel for the result of the model). We compare this figure with the data (Fig 3, right panel), where mothers are ranked according to their fluorescence, a substitute for their plasmid copy number. The fluorescence (≈ plasmid copy number) of both daughters are drawn over the mother’s rank. If the mothers’ rank is small, we find one single, linearly increasing branch formed by both daughters. At rank 100, suddenly a bifurcation takes place, where the lower branch stays approximately at a constant level, while the upper branch steeply increases. Qualitatively, the two panels in Fig 3 agree very well. The theoretical ESSS describes the experimental outcome appropriately.

### Conceptual model

In order to better understand the mechanism that yields the observed ESSS, we discuss an oversimplified, conceptual model. Hereby we take up ideas about damage inheritance [26]. Let us assume that a “newborn” cell incorporates *z*_0_ plasmids. We neglect the effect of plasmid accumulation during the lifetime on the division rate. In our conceptional model, the division rate is simply defined by $\beta_{0}\left(1-z_{0}/\hat{z}\right)$. The fitness *f*_0_ of the cell is identical with this division rate,

$$\begin{array}{c} f_{0}=\beta_{0}\left(1-z_{0}/\hat{z}\right). \end{array}$$

To make the argument as clear as possible we consider a deterministic model. The time *T* to the next division is given by the deterministic value

$$\begin{array}{c} T\left(z_{0}\right)=\frac{1}{\beta_{0}\left(1-z_{0}/\hat{z}\right)}. \end{array}$$

**Fig 4:**
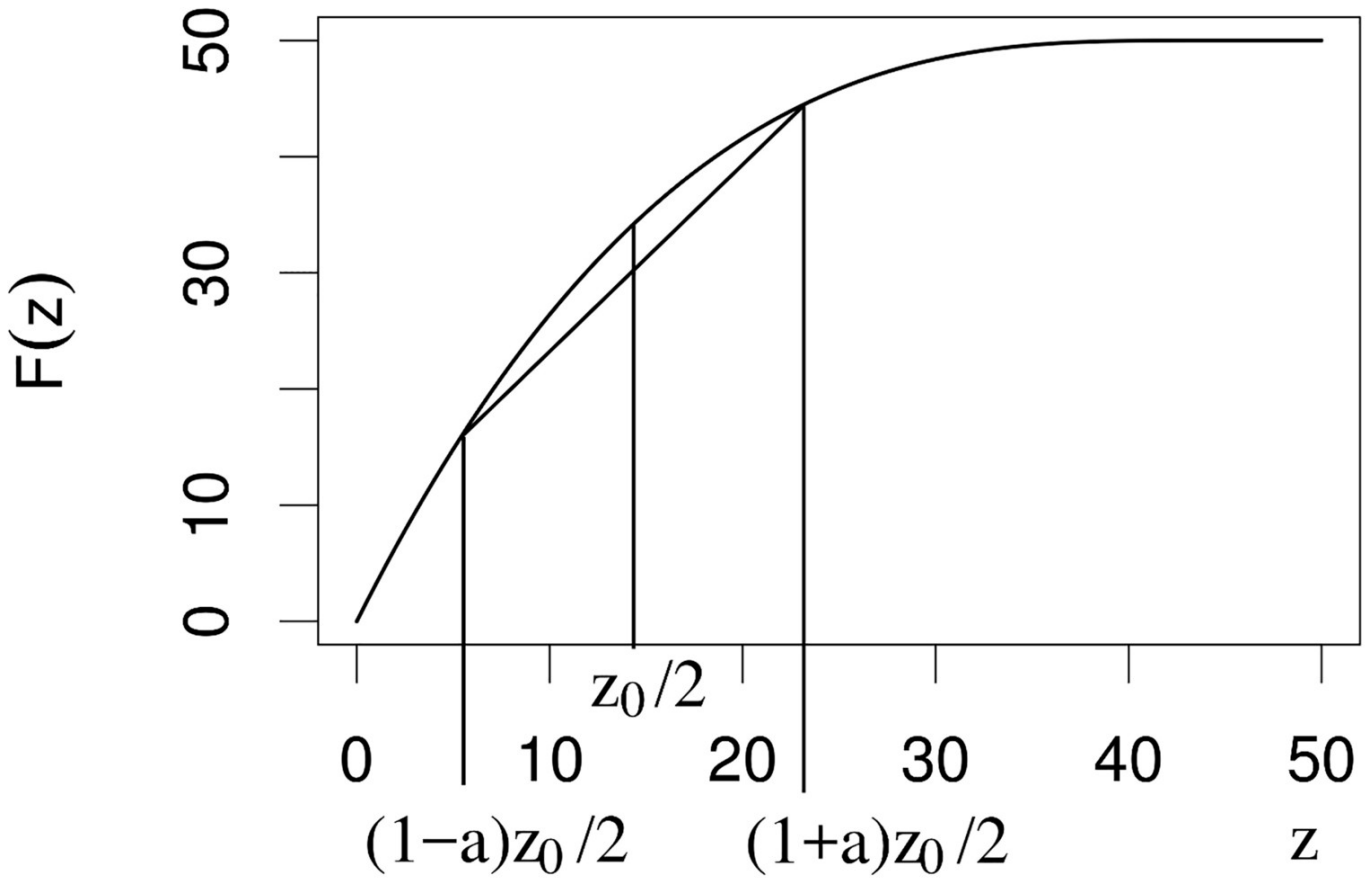
Visualization of *F*(*z*) in the conceptual model. *F*(*z*) denotes the copy number of a cell right before division that did start with *z* (*β*_0_ = *b* = 1/*h*, $\hat{z}=50$). As *F*(*z*) is concave, *F*(*z*/2) > {*F*((1 + *a*)*z*/2) + *F*((1 − *a*)*z*/2)}/2.

During this time, plasmids accumulate according to $z^{\prime }\left(t\right)=bz\left(t\right)\left(1-z\left(t\right)/\hat{z}\right)$, *z*(0) = *z*_0_. The plasmid copy number just before the next cell division amounts to *F*(*z*_0_) = *z*(*T*_0_(*z*_0_)), that is [44]

$$\begin{array}{c} F\left(z_{0}\right)=\frac{z_{0}\;\hat{z}}{e^{-\left(b/\beta_{0}\right)/\left(1-z_{0}/\hat{z}\right)}\left(\hat{z}-z_{0}\right)+z_{0}} \end{array}$$

(see Fig 4 for a graph of *F*(*z*_0_)). At time *T*(*z*_0_) our cell divides, and has two progeny cells. In case of equal segregation, both daughters receive the same number of plasmids, and hence the fitness of both daughters is

$$\begin{array}{c} f_{1}=\beta_{0}(1-\frac{1}{2\;\hat{z}}F\left(z_{0}\right)). \end{array}$$

Note that $F\left(z_{0}\right)\leq \hat{z}$, such that the fitness is always positive. Unequal segregation can be expressed by an additional parameter *a* ∈ (0, 1], where one daughter recieves (1 + *a*)*F*(*z*_0_)/2, and the other (1 − *a*)*F*(*z*_0_)/2 plasmids. In this case, the fitnesses of the daughters are given by

$$\begin{array}{c} f_{\pm }=\beta_{0}(1-\frac{1}{2\;\hat{z}}\;\left(1\pm a\right)\;F\left(z_{0}\right)). \end{array}$$

As the fitness is linear in the plasmid load, the average fitness ${\bar{f}}_{1}=\left(f_{+}+f_{-}\right)/2$ in the first generation is not changed by asymmetric segregation, ${\bar{f}}_{1}=f_{1}$. There seems to be no reason for the advantage of asymmetric segregation. However, plasmids come with a metabolic burden—though the average fitness is unchanged, the quality of the two daughters is different. One of the daughters starts her life with a smaller metabolic burden than her sister. This becomes visible in the *second* generation: Let *z*_1_ = *F*(*z*_0_), the copy numbers of the four progeny cells are given by

$$\begin{array}{c} \frac{1}{2}\left(1\pm a\right)\;F(\left(1\pm a\right)z_{1}/2). \end{array}$$

That is, the average fitness in the second generation reads

$$\begin{array}{c} {\bar{f}}_{2}=\beta_{0}(1-\frac{1}{2\;\hat{z}}\;\;\frac{1}{2}[F\left(\left(1+a\right)z_{1}/2\right)+F\left(\left(1-a\right)z_{1}/2\right)]). \end{array}$$

As *F*(*x*) is concave (see Fig 4), *F*(*z*_1_/2) > [*F*((1 + *a*)*z*_1_/2) + *F*((1 − *a*)*z*_1_/2)]/2. The fitness in the second generation is larger for asymmetric than for symmetric segregation. Plasmids are dumped in one daughter (seriously decreasing her fitness) to allow the other daughter to reproduce efficiently.

The argument so far considered plasmids exclusively as a burden, and thus we could use the ideas about damage distribution developed by Chao [26]. However, cells also benefit from plasmids as they protect against antibiotics. The fitness of a cell without plasmids is strongly decreased. If *z*_0_ is large, there is no risk that the plasmids are lost in the next few generations, also in case of (moderate) asymmetric segregation. This is different if *z*_0_ is small. We introduce a factor *q*(*z*) ∈ [0, 1] that express the relative fitness reduction by plasmid loss for a cell (or part of her progeny) that starts with *z* plasmids. “Relative” does refer to a hypothetical case where also cells without any plasmids are perfectly protected against antibiotics. Obviously, *q*(0)≈0, and *q*(*z*)→1 if *z* becomes large. In our conceptual model, we define *q*(*z*) = *z*/(*K*+ *z*), where *K* is the copy number *z* necessary to achieve *q*(*z*) = 1/2. Under these circumstances, we find for the average fitness in the first generation (using the notation introduced above)

$$\begin{array}{c} {\bar{f}}_{1}=\frac{1}{2}(q\left(\left(1+a\right)z_{1}/2\right)\;f_{+}+q\left(\left(1-a\right)z_{1}/2\right)\;f_{-}). \end{array}$$

Note that *q*(*z*) is increasing, while its derivative is decreasing. That is, *q*((1 + *a*)*z*_1_/2) increases slower in *a* than *q*((1 − *a*)*z*_1_/2) decreases. Therefore, the stronger reduction of the larger fitness *f*_−_ cannot be compensated by the weaker reduction of the smaller fitness *f*_+_. It is not a good idea to push one cell towards plasmid loss, though this cell would have (without the disadvantage at total plasmid loss) the better fitness. More formally, a straightforward computation based on the monotonicity properties of *q*(*z*) and *q*′(*z*) shows that the derivative of ${\bar{f}}_{1}$ with respect to *a* is negative for *a* > 0 (see S1 Text, monotonicity of the fitness in *a*), and hence the fitness is maximized in the symmetric case (*a* = 0). The beneficial effects of plasmids force the cell to use symmetric segregation if the copy number is small.

In that discussion, we only considered the average fitness of one or two generations. The argument is only complete if we follow the average fitness over many generations. This task is accomplished in the full model, with results in line with our conceptual approach.

## Discussion

The present study concentrated on the question of how evolutionary forces influence plasmid segregation strategies for high copy plasmids. Therefore, a model for a bacterial population, carrying a high copy plasmid that may segregate unequal has been developed, and Adaptive Dynamics has been used to determine the ESSS. The plasmid we have in mind carries genes encoding for an antibiotic resistance. It turns out that in case of few plasmids, each daughter receives in average an equal share of plasmids (equal segregation). For larger copy numbers, one daughter receives systematically more; the model predicts a distinctively unequal segregation strategy (up to 20% plasmids for one, and 80% plasmids for the other daughter). This finding is rather stable w.r.t. variation of parameters, the augmentation of the model by horizontal plasmid transfer (S1 Text, effect of horizontal plasmid transmission), or switching environment (section “Long-term behavior”). The ESSS determined by numerical analysis is in line with earlier experimental observations [11, 15].

We can understand the ESSS intuitively. In the case of few plasmids, the probability that each of the daughters receives at least one plasmids is maximized. For low-copy plasmids, there is a biochemical mechanism controlling the plasmid segregation [9–11]. In the present case of high copy plasmids, there is no such control [12–14], such that the ESSS is the best the bacteria can do to prevent that a daughter does not inherit any plasmid and is unprotected.

If, however, the copy number is high, plasmids become a burden and the bacterial reproduction rate is decreased [18]. In this situation, it is better to dump many plasmids into one daughter. Consequently, that daughter grows only slowly or even stops to divide, while the other daughter is in the preferred range of plasmid copy numbers: small enough to not be a metabolic burden, and high enough such that both of her daughter cells receive plasmids. And indeed, the numerical results reveal that the average copy number of one daughter is kept fairly constant, independently on the mothers’ copy number, while consequently the average copy number of the second daughter increases; also in the experimental data [15], we could find back this structure (Fig 3, right panel). The molecular mechanisms for the realization of the ESSS surely represent a major metabolic burden. Most likely, the energetic costs for the DNA synthesis accompanying the accumulation of plasmids are high. Moreover, a competition between chromosomal and plasmid replication might occur. This might explain, that cells full with plasmids simply stop to divide, their fitness tends to zero. In contrast, we expect that the fitness costs for the formation of the molecular segregation apparatus itself will only play a minor role.

It is interesting that our finding is in line with experimental results for waste proteins in cells [45]. Plasmids in high numbers represent mainly a burden for the cell, resembling cell damage. And indeed, the theory of cell damage suggest that unequal segregation increases the average fitness of the population [26, 46]. Bacteria accumulate proteins that are not recycled but are waste. To prohibit poisoning by these old molecules, bacteria tend to dump them into one daughter. The explanation is similar to the explanation we developed to understand plasmid segregation: one daughter is sacrificed in order to allow the other daughter to reproduce at the maximal rate.

We may interpret the ESSS as a special form of division of work. Though we do not find clearly separated subpopulations, we may nevertheless identify three different phenotypes: (a) one phenotype without plasmids, specialized to the antibiotics-free environment (b) one phenotype with few plasmids, specialized for the environment with antibiotics, and (c) the phenotype with many plasmids, serving as a rubbish-heap, and perhaps also as a protein source for the remaining population (once these cells dissolve). In this point of view, the underlying evolutionary mechanism shaping the plasmid segregation strategy is the same as that leading to phenotypic heterogeneity as persister cells [30], sporulation [47] and competence [48].

## Models

### Model

We have a population of cells with a heterogeneous plasmid copy number distribution. There are advantageous and disadvantageous aspects of the plasmids for a cell. The advantage is the protection against antibiotics, while the disadvantage is decreased growth rate due to the metabolic burden. An overview of symbols and parameter values is given in Table 1. The rationale for the choice of the parameters is explained below.

**Table 1:** Symbols and parameters used in the model. Please find the rational for the parameter choices section “Parameters”.

Symbol	meaning	value no antibiotics	value with antibiotics
*z*	copy number of a cell		
*u*_*z*_(*t*)	population size, dependent on time and *z*		
$\hat{z}$	maximal copy number (plasmids)	50	50
*b*	logistic growth parameter (plasmids)	1.2/*h*	1.2/*h*
*β* (*z* = 0)	bacterial reproduction rate	1/*h*	0
*β* (*z* > 0)	bacterial reproduction rate	$1\;{\left(1-\;z/\hat{z}\right)}_{+}/h$	$1\;{\left(1-\;z/\hat{z}\right)}_{+}/h$
*μ* (*z* = 0)	bacterial death rate	0	5/*h*
*μ* (*z* > 0)	bacterial death rate	0	0
*p*_*z*_	segregation probability, dependent on the mothers’ copy number *z*	varied	

We model two different levels:

1. Reproduction of plasmids within a cell, and
2. Growth of cells in dependence of plasmid content and environment.

#### State of the system

We structure the population by the number of plasmids,

$$\begin{array}{c} u_{z}\left(t\right)=\text{amout}\;\text{of}\;\text{cells}\;\text{in}\;\text{the}\;\text{population}\;\text{with}\;z\;\text{plasmids},z\in N_{0}. \end{array}$$

Apart from the structured population, we also need to know if antibiotics are present. Let *α* = 0 for no antibiotics, and *α* = 1 if antibiotics are in the environment. We will not model the intrinsic dynamics of this concentration but assume that the time course *α* = *α*(*t*) is given.

#### Dynamical process I: Replication of plasmids between two cell divisions

The plasmids within a cell follow a logistic birth process. Consider a fixed cell with *z* plasmids. The plasmids reproduce within this cell at rate $bz\;(1-z/\hat{z})$, where $\hat{z}\in N$ denotes the maximum number of plasmids. The dynamics of *u*_*z*_(*t*) is described by the master equations for this birth process for the plasmids,

$$\begin{array}{c} {\dot{u}}_{z}=-\;bz\left(1-z/\hat{z}\right)\;\;u_{z}+b\left(z-1\right)\left(1-\left(z-1\right)/\hat{z}\right)\;\;u_{z-1},\;z=0,\ldots ,\hat{z} \end{array}$$

where we formally define *u*_−1_(*t*) = 0.

#### Dynamical process II: Cell divisions

Now we come to the second aspect: cell division. The division rate of a cell depends on the number of plasmids *z* (due to the metabolic burden), and the presence of antibiotics indicated by *α*,

$$\begin{array}{c} \beta =\beta (z,\alpha ). \end{array}$$

Cells without plasmids reproduce in absence of antibiotics at rate *β*(0, 0) = *β*_0_, in presence of antibiotics we assume that reproduction is impossible, *β*(0, 1) = 0. We furthermore assume that bacteria carrying at least one plasmid are resistant, and have the same reproduction rate with and without antibiotics in the environment. For *z* > 0, we may write *β*(*α*, *z*) = *β*(*z*). Measurements indicate that the copy number decreases the fitness of cells approximately linearly [49], where one plasmid accounts for a reduction of the fitness (reproduction rate) by 0.17% [49] to 0.5% ([50], table page 250). Experiments [15] did show that cells with a high plasmid content do not divide anymore. Thus we choose to model the effect of the metabolic burden in a linear way, such that the bacterial reproduction rate for copy number $z=\hat{z}$ becomes zero,

$$\begin{array}{c} \beta \left(z\right)=\beta_{0}\left(1-z/\hat{z}\right). \end{array}$$ (1)

High copy plasmids are present in copy numbers between 50 and 800, such that this formula indicates in line with a reduction of fitness per plasmid by $100/\hat{z}\%\approx 0.125\%$ (800 plasmids)—2% (50 plasmids). That is, the decrease of the fitness by plasmids is within the range of the experimentally observed values.

We note that the plasmid reproduction rate, as well as the bacterial reproduction rate, are scaled by $1-z/\hat{z}$: Biomass production is inhibited by the metabolic burden due to the plasmids. Hence the metabolic burden affects the cell division rate in the same way as plasmid production rate.

In the case of cell division, first of all, two cells are produced out of one cell. Each of the cells inherits some plasmids. The number of the plasmids of the two daughter cells (at birth) add up to the number of plasmids of the mother cell. Select randomly one of the two daughter cells. Each plasmid has the probability *p* to move into the selected daughter cell. If *p* = 0.5, the distribution is symmetric, in average each daughter receives half of the plasmids. If *p* = 0, all plasmids move in one daughter while the other receives nothing. The segregation is maximally unsymmetrical.

The probability *p* depends on the mothers’ plasmid copy number *z*, that is, *p* = *p*_*z*_. The assumptions above imply that plasmid segregation happens according to a Binomial distribution,

$$\begin{array}{ll} g\left(z;z_{0}\right) & =P(z\;\text{plasmids}\;\text{contained}\;\text{in}\;\text{the}\;\text{selected}\;\text{cell}|\text{mother}\;\text{contained}\;z_{0}\;\text{plasmids})\\ & =(\begin{array}{c} z_{0}\\ z \end{array})p_{z_{0}}^{z}{\left(1-p_{z_{0}}\right)}^{z_{0}-z}. \end{array}$$ (2)

Our model reads

$$\begin{array}{ccc} {\dot{u}}_{z} & = & -\;bz\left(1-z/\hat{z}\right)\;\;u_{z}+b\left(z-1\right)\left(1-\left(z-1\right)/\hat{z}\right)\;\;u_{z-1}\\ & & -\beta \left(z,\alpha \right)u_{z}+\sum_{z_{0}=z}^{\hat{z}}\left[g\left(z;z_{0}\right)+g\left(z_{0}-z;z_{0}\right)\right]\beta \left(z_{0},\alpha \right)u_{z_{0}}. \end{array}$$ (3)

In the paper we focus on the segregation of plasmids. We therefore define the segregation kernel as the distribution of plasmids in both daughters.

**Definition 1:** *The segregation kernel for a mother cell with z*_0_ *plasmids is defined as*

$$\begin{array}{c} k\left(z;z_{0}\right)=g\left(z;z_{0}\right)+g\left(z_{0}-z;z_{0}\right). \end{array}$$

Note that the segregation kernel inherits a certain symmetry, *k*(*z*;*z*_0_) = *k*(*z*_0_ − *z*; *z*_0_).

#### Dynamical process III: Cell death

In presence of antibiotics, the cells may die. We have a mortality *μ* = *μ*(*z*, *α*). All in all, we obtain the model

$$\begin{array}{ccc} {\dot{u}}_{z} & = & -\;bz\left(1-z/\hat{z}\right)\;\;u_{z}+b\left(z-1\right)\left(1-\left(z-1\right)/\hat{z}\right)\;\;u_{z-1}\\ & & -\beta \left(z,\alpha \right)u_{z}+\sum_{z_{0}=z}^{\hat{z}}\left[g\left(z;z_{0}\right)+g\left(z_{0}-z;z_{0}\right)\right]\beta \left(z_{0},\alpha \right)u_{z_{0}}-\mu \left(z,\alpha \right)u_{z}. \end{array}$$ (4)

Without antibiotics, cells will not die, *μ*(0, *z*) = 0. With antibiotics, cells inheriting plasmids are protected and will also not die (*μ*(1, *z*) = 0 for *z* > 0). Only cells without plasmids die in presence of antibiotics at rate *μ*_0_, *μ*(1, 0) = *μ*_0_ > 0.

Please note that our model is linear. If we introduce the subpopulation without plasmids *x* = *u*_0_(*t*) and the structured subpopulation carrying plasmids *y* (vector), *y*_*z*_(*t*) = *u*_*z*_(*t*), $z=1,\ldots ,\hat{z}$, we may write the system (4) by

$$\begin{array}{ccc} x_{0}^{\prime } & = & \left(\beta \left(0,\alpha \right)-\mu \left(0,\alpha \right)\right)x_{0}\left(t\right)+B^{T}y\left(t\right) \end{array}$$ (5)

$$\begin{array}{ccc} y^{\prime } & = & A\;y \end{array}$$ (6)

where *B* is a vector and *A* a matrix defined by Eq (3); *B* and *A* depend on the segregation strategy ${\left(p_{z}\right)}_{z=1,\ldots ,\hat{z}}$, but not on the environment.

### Long-term behavior and ESSS

The linear system is not irreducible [44]. We have one exponentially growing solution without plasmids (*y* = 0), and one exponentially growing solution with plasmid-bearing cells (*y* > 0). Let us first consider the plasmid-free solution (*y* = 0). We may introduce the average growth rate in the switching environment

$$\begin{array}{c} \lambda_{0}\left(t\right)=\frac{1}{t}\int_{0}^{t}\left(\beta \left(0,\alpha \left(\tau \right)\right)-\mu \left(0,\alpha \left(\tau \right)\right)\right)\;d\tau \end{array}$$

and write this solution as

$$\begin{array}{ccc} x_{0}\left(t\right) & = & x_{0}\;e^{t\lambda_{0}\left(t\right)},\;y\left(t\right)=0. \end{array}$$ (7)

Now we turn to the second solution (*y* > 0). If plasmids are present, the linear ODE *y*′ = *Ay* (which is irreducible) tends to the dominating solution of that ODE. The spectral bound λ_1_ (the eigenvalue with the largest real part) of *A* is real, and has a positive eigenvector $\hat{y}$, such that $A\hat{y}=\lambda_{1}\hat{y}$. As *A* depends on the segregation strategy ${\left(p_{z}\right)}_{z=1,\ldots ,\hat{z}}$, also this eigenvalue is a function of *p*_*z*_, λ_1_ = λ_1_(*p*_*z*_). In the long run, the solution for *y*(*t*) reads [44]

$$\begin{array}{c} y\left(t\right)=c\;e^{\lambda_{1}\;t}\hat{y} \end{array}$$ (8)

for some positive constant *c*. Let us assume that $y\left(0\right)=\hat{y}$. The variation-of-constant formula yields

$$\begin{array}{c} x\left(t\right)=x_{0}\;e^{t\lambda_{0}\left(t\right)}+\;\int_{0}^{t}e^{\int_{s}^{t}\left(\beta \left(0,\alpha \left(\tau \right)\right)-\mu \left(0,\alpha \left(\tau \right)\right)\right)\;d\tau }\;B^{T}\hat{y}e^{\lambda_{1}\;s}\;ds,\;y\left(t\right)=c\;e^{\lambda_{1}\;t}\hat{y}. \end{array}$$ (9)

As an immediate consequence, we have the following theorem.

**Theorem 1:** *Let* $N\left(t\right)=x_{0}\left(t\right)+\sum_{z=1}^{\hat{z}}y_{z}\left(t\right)$. *Assume that the limit* $\lim_{t\to \infty }\lambda_{0}\left(t\right)={\hat{\lambda }}_{0}$ exists and ${\hat{\lambda }}_{0}>\lambda_{1}$, then $\sum_{z=1}^{\hat{z}}y_{z}/N\left(t\right)\to 0$ *and the plasmid is lost by the population. If* ${\hat{\lambda }}_{0}<\lambda_{1}$, *then* $y\left(t\right)/N\left(t\right)\to c\hat{y}$ *for some c > 0, and we find a stable plasmid bearing subpopulation*.

In order to define the ESSS, we consider two competing subpopulations *y* and $\hat{y}$ with different plasmid segregation characteristics *p*_*z*_ resp. ${\hat{p}}_{z}$. If $\lambda_{1}\left(p_{z}\right)>\lambda_{1}\left({\hat{p}}_{z}\right)$, then *y* will grow faster then $\hat{y}$ and out-compete $\hat{y}$. The evolutionary stable segregation strategy maximizes λ_1_(*z*).

**Definition 2:** *The plasmid segregation strategy* $p_{z}^{*}\in {\left[0,0.5\right]}^{\hat{z}}$ *that maximizes λ*_1_(*p*_*z*_) *is called the ESSS*.

Note that the ESSS $p_{z}^{*}$ is only sensible if the plasmid is not lost by the population, that is, if ${\hat{\lambda }}_{0}<\lambda_{1}\left(p_{z}^{*}\right)$. Under this condition, the population with plasmid segregation strategy $p_{z}^{*}$ cannot be invaded by another segregation strategy. Adaptive Dynamics [34] predict that evolutionary forces drive the segregation strategy towards $p_{z}^{*}$. It is straightforward to extend this approach and to investigate a fluctuating environment (see section “Long-term behavior”).

### Numerical procedures

It is not possible to analytically compute the ESSS. We use numerical analysis to find $p_{z}^{*}$ by means of the steepest ascent method [51]. Let ∇ denote the gradient w.r.t. $p_{z}=\left(p_{1},\ldots ,p_{\hat{z}}\right)$. We introduce the artificial time *s* (note that *s* has nothing in common with chronological time *t*, but basically measures the time we already spend using the optimization method), and consider *p*_*z*_ = *p*_*z*_(*s*), the segregation strategy as a function of this artificial time *s*. In order to maximize λ_1_(*p*_*z*_), we solve the ODE

$$\begin{array}{c} \frac{d}{ds}p_{z}\left(s\right)=\nabla \lambda_{1}\left(p_{z}\left(s\right)\right). \end{array}$$

Assume that this ODE converges to $p_{z}^{*}$. Then, $p_{z}^{*}$ is a stationary state of the ODE, and $\nabla \lambda_{1}\left(p_{z}^{*}\right)=0$. That is, we obtain a candidate for a (local) maximum, minimum, or saddle point. We ensure that we have a local maximum by varying *p*_*z*_ locally around $p_{z}^{*}$ and inspecting λ_1_(*p*_*z*_). Next, we need to check if we have not only a local but a global maximum. This is, strictly spoken, impossible. In order to ensure that we have at least most likely a global maximum, we use different initial conditions and check that we always end up in the same maximum $p_{z}^{*}$.

### Parameters and results of the sensitivity analysis

In order to determine the ESSS, we only need to consider the subpopulation *y*. The dynamics of this subpopulation is solely influenced by $\beta_{0}\left(1-z/\hat{z}\right)$, and $b(1-z/\hat{z})$, that is, by the parameters *β*_0_, *b*, and $\hat{z}$. Since rescaling time does not change the dynamics, we may use without restriction *β*_0_ = 1/*h*. It turns out that the ESSS assumes the form observed in data (Fig 3) if *b* is at least in the range of *β*_0_ or larger. We choose *b* = 1.2/*h*. For this parameter, the ESSS closely resembles the observed structure. Analysis of the experiment in [15] yields *β*_0_ ≈ *b* ≈ 1/*h* (see [52], Fig. 6). Note that evolutionary forces most likely shaped the plasmid segregation strategy for the wild-type plasmid, while we use here a biotechnological tailored variant, that e.g. produces GFP. As the GFP production will influence the plasmid reproduction, it is rather possible (but not proven) that the wild-type form of the plasmid grows slightly faster than the variant used here.

Sensitivity analysis (S1 Text, sensitivity analysis) indicates that the results are stable w.r.t. parameter variation in a reasonable range. The carrying capacity of the plasmids $\hat{z}$ has almost no influence (S1 Text, variation of the carrying capacity of plasmids). Neither the horizontal plasmid transfer (S1 Text, effect of horizontal plasmid transmission) nor a fluctuating environment does change the ESSS. Two main ingredients are necessary: First, the plasmids need to come with a reasonable metabolic burden. If the metabolic burden is only decreased by 20%, the segregation strategy of the ESSS is always equal (S1 Text, scaling the metabolic burden). This observation is intuitive, as the burden forces unequal segregation to be favorable (see also section “conceptual model” above). The second ingredient is the growth rate of plasmids *b*, that should be at least in the same range or faster than the (maximal) division rate of cells *β*_0_ (S1 Text, variation of the reproduction rate of plasmids). We can also understand this observation intuitively: only if cells start to accumulate at the carrying capacity $\hat{z}$, the segregation strategy is adapted to prevent that all daughters eventually will be filled up with plasmids. Recalling Fig 1, we find that division of cells counteracts plasmid aggregation. If cell division happens at a higher rate than plasmid division (*β*_0_ > *b*), the cells do not tend to accumulate plasmids, and hence an equal segregation is the ESSS.

## Supporting information

S1 Text Sensitivity analysis, effect of horizontal plasmid transmission, and monotonicity of the fitness in *a*. (PDF)

S1 Data Single cell data. (ZIP)
