# Supplementary material for: Evolutionary model for the unequal segregation of high copy plasmids
Source: PLoS Comput Biol. 2019 Mar 5;15(3):e1006724. doi: 10.1371/journal.pcbi.1006724 (PMC6420036; doi:10.1371/journal.pcbi.1006724)
Supplement: S1 Text — (PDF) [file pcbi.1006724.s001.pdf]

Evolutionary model for the unequal segregation of high copy plasmids

Karin Münch, Richard Münch, Rebekka Biedendieck, Dieter Jahn, Johannes Müller

Supplementary Informations

Sensitivity analysis

We vary parameters to test our model for stability, respectively to identify the sensitivity of the ESSS with respect to the variation of the parameters.

As noted before, the ESSS is invariant under rescaling of time. We thus fix the reproduction rate of bacteria, $\beta_0 = 1/h$. The other values used as reference are $b = 1.0/h$ and $\hat{z} = 50$ (see section 1.1.1 for the figures with these parameter values).

Variation of the reproduction rate of plasmids

We test $b = 0.8/h$, $b = 0.9/h$, $b = 1.0/h$, $b = 1.5/h$, and $b = 2/h$. We present for each parameter set the ESSS (p_z), the copy number distribution (population density over copy number) and the average copy number in both daughters over the mothers' copy number.

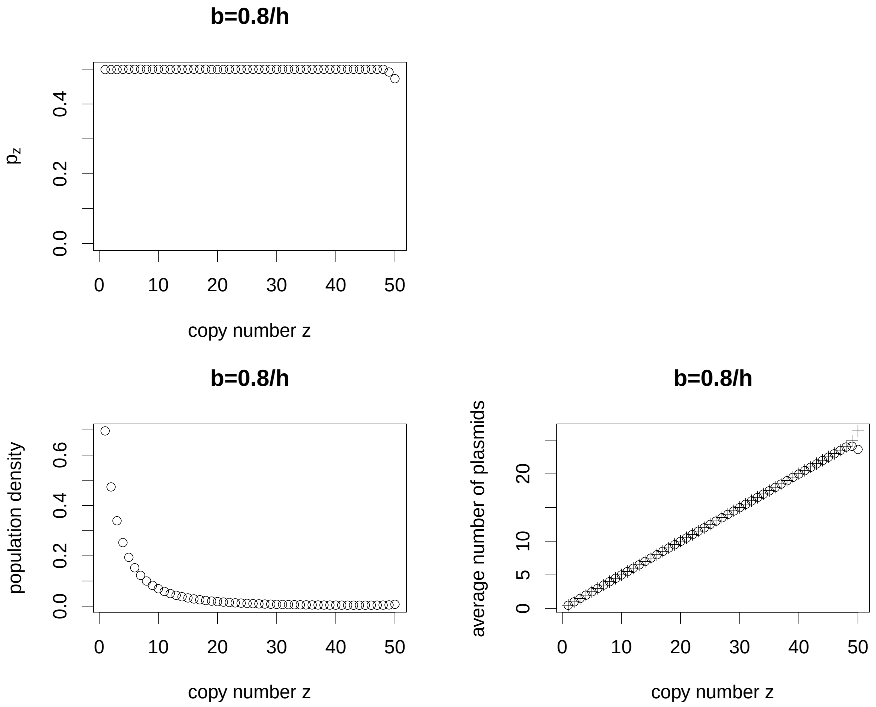

Figure A: Parameters: $\beta_0 = 1/h$, $\hat{z} = 50$, and $b = 0.8$.

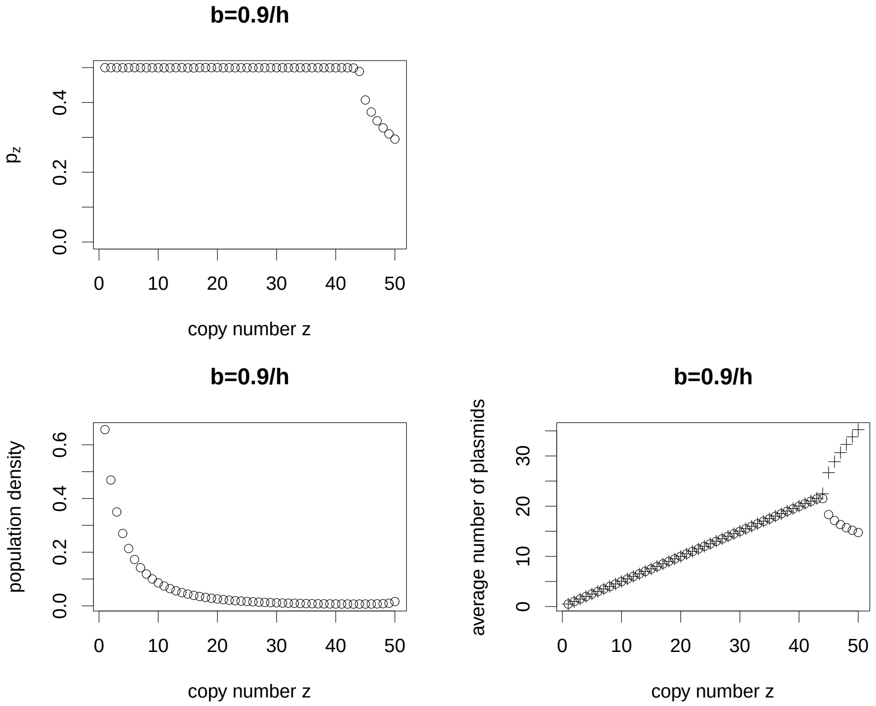

Figure B: Parameters: $\beta_0 = 1/h$, $\hat{z} = 50$, and $b = 0.9$.

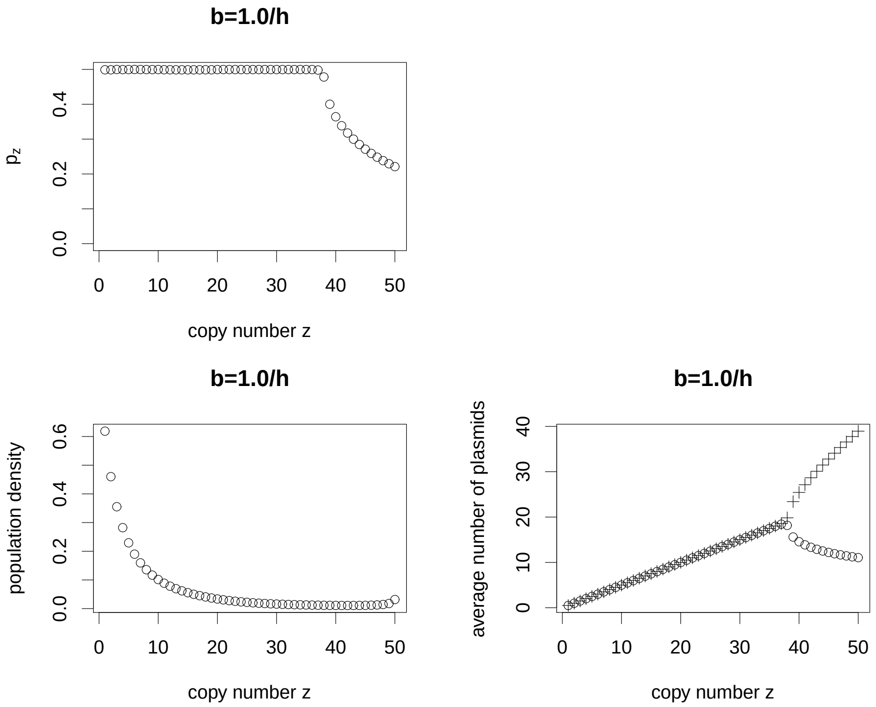

Figure C: Parameters: $\beta_0 = 1/h$, $\hat{z} = 50$, and $b = 1.0$.

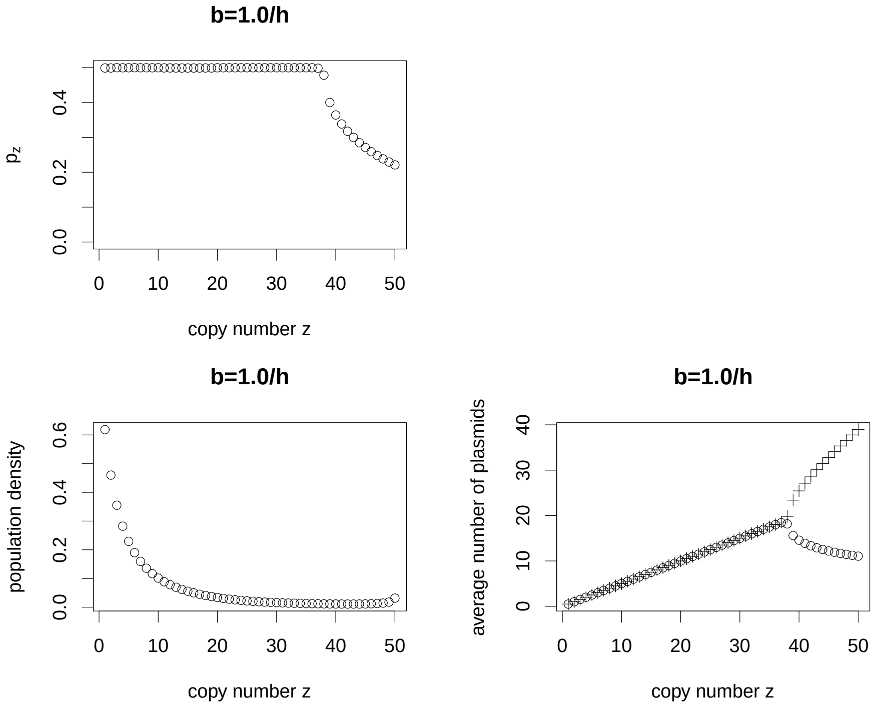

Figure D: Parameters: $\beta_0 = 1/h$, $\hat{z} = 50$, and $b = 1.0$.

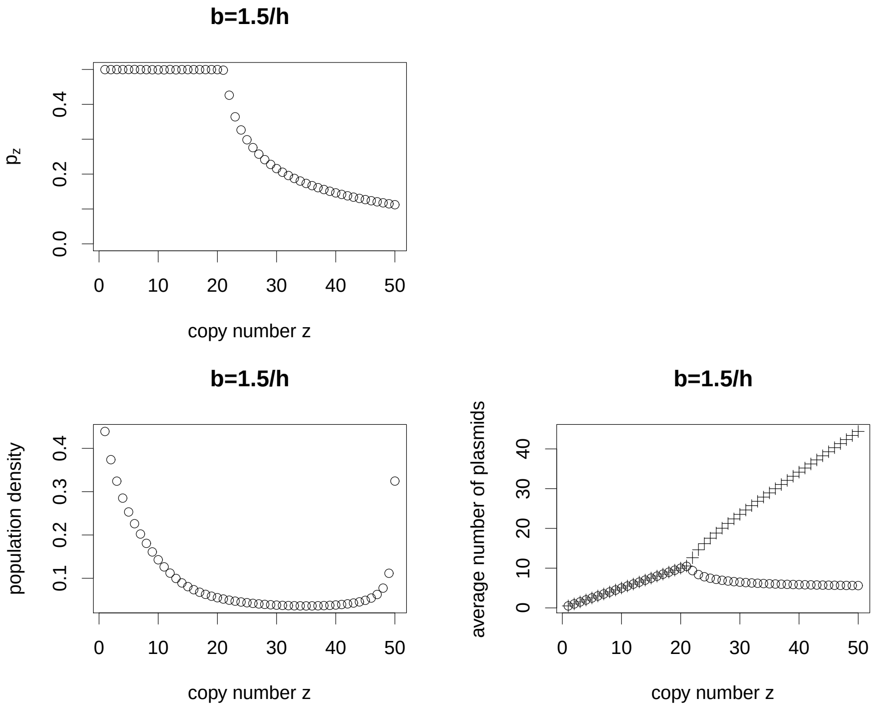

Figure E: Parameters: $\beta_0 = 1/h$, $\hat{z} = 50$, and $b = 1.5$.

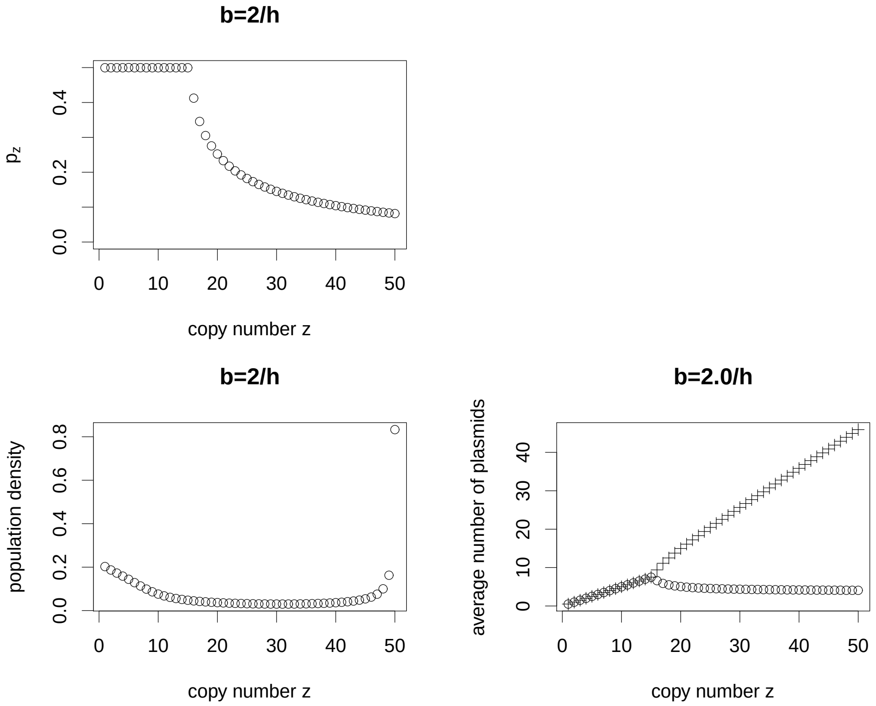

Figure F: Parameters: $\beta_0 = 1/h$, $\hat{z} = 50$, and $b = 2.0$.

If $b \approx 0.9/h$ or larger, we find unequal segregation pattern to appear (Fig. 1-6). If b is too small (in comparison with β), equal segregation is optimal. Only for those values, cell division is not able to If b is distinctively smaller than β_0, bacterial division is able to effectively counteract plasmid reproduction, and the population does not accumulates at $z = \hat{z}$. In this case, equal segregation is best.

Variation of the carrying capacity of plasmids

We test $\hat{z} = 100$ and $\hat{z} = 200$.

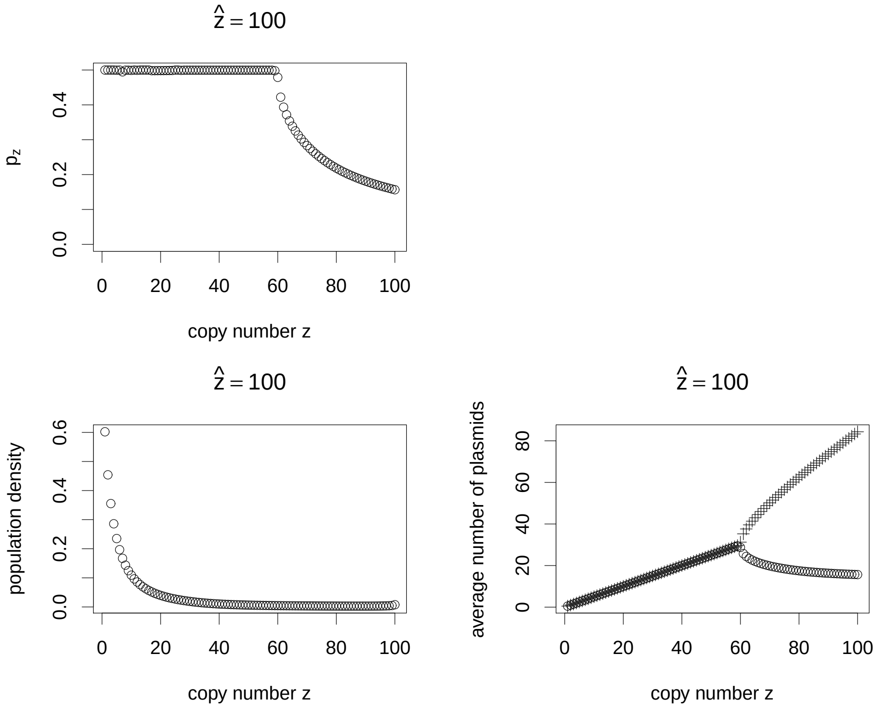

Figure G: Parameters: $\beta_0 = 1/h$, $b = 1.0/h$, and $\hat{z} = 100$.

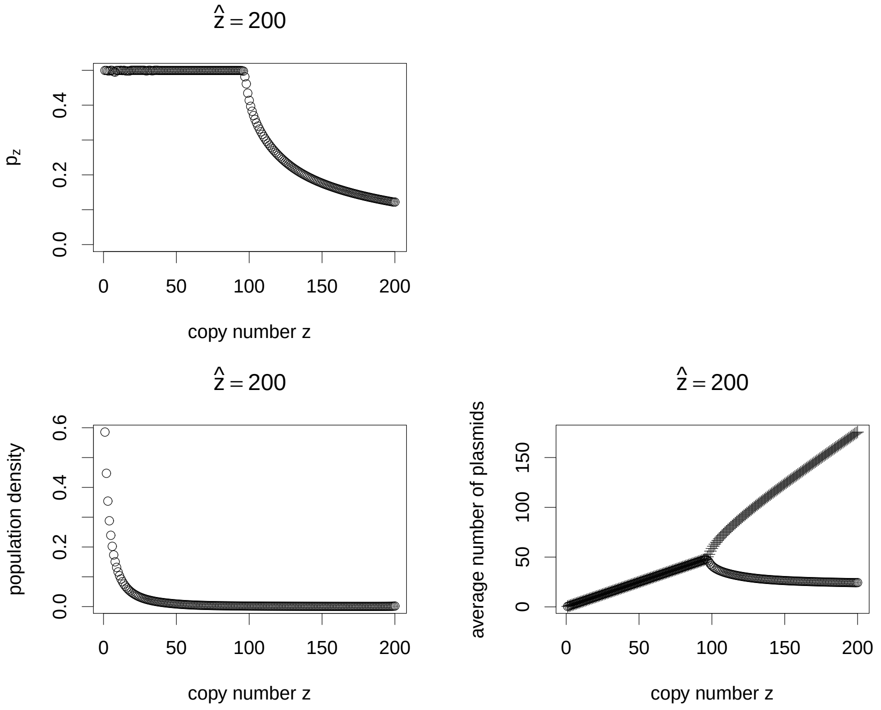

Figure H: Parameters: $\beta_0 = 1/h$, $b = 1.0/h$, and $\hat{z} = 200$.

The ESSS scales approximately linearly with $\hat{z}$ (rescaling the x-axis for $\hat{z} = 100$ by factor 2 almost yields the figure for $\hat{z} = 200$, see Fig. 7, 8).

Scaling the metabolic burden

In the model we always assume that the cell reproduction rate becomes zero for $z = \hat{z}$. We now consider

$$\beta(z) = \beta_0(1 - \theta\, z/\hat{z}),$$

such that we have the original model for $\theta = 1$. If $\theta < 1$, the metabolic burden per plasmid is decreased and cells are still able to divide, even for $z = \hat{z}$. We consider $\theta = 0.9$ and $\theta = 0.8$.

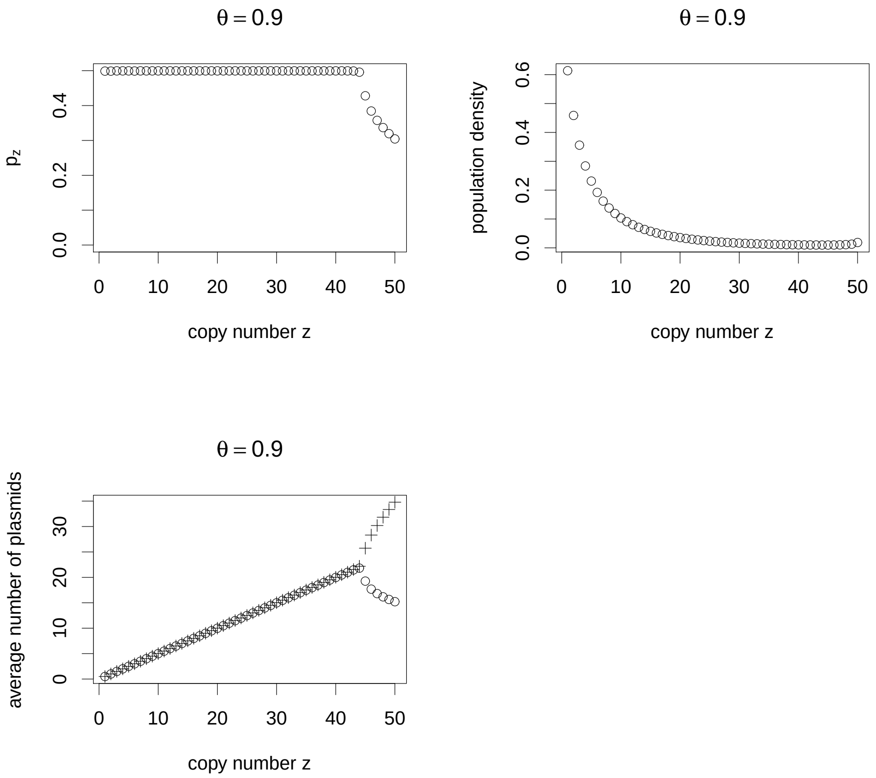

Figure I: Parameters: $\beta_0 = 1/h$, $b = 1/h$, $\hat{z} = 50$, and $\theta = 0.9$

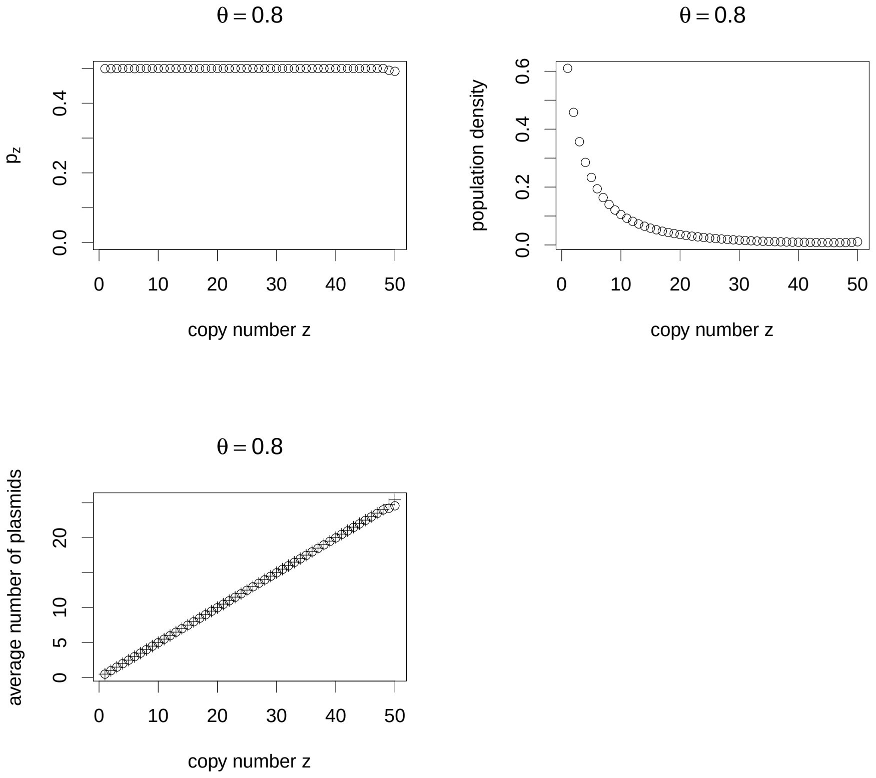

Figure J: Parameters: $\beta_0 = 1/h$, $b = 1/h$, $\hat{z} = 50$, and $\theta = 0.8$

If the metabolic burden is only 80% of the chosen one (where cells with copy number $\hat{z}$ cannot divide any more) leads to an equal segregation behavior. Unequal segregation only becomes an ESSS if the metabolic burden is heavy for $z = \hat{z}$ (Fig. 9, 10).

Effect of horizontal plasmid transmission

We extend the model by horizontal transmission of plasmids from cell to cell (e.g. by conjugation) to ensure that our conclusion are stable. The relevance of this transmission is rather unclear, the rates estimated in literature range from $\approx 10^{-10}/(h \text{ cell})$ [1], $4.2\ 10^{-8}/(h \text{ cell})$ [2] to $2.5\ 10^{-11}/(h \text{ cell})$ [3].
However, it might happen that this effect is of importance in the long run. Therefore it is sensible to check the influence of horizontal plasmid transmission. We repeat the complete modeling process, including the horizontal plasmid transmission.

Dynamical process I: Replication of plasmids between two cell divisions. The plasmids within a cell follow a logistic birth process. Consider a fixed cell with z plasmids. The plasmids reproduce within this cell at rate $bz(1 - z/\hat{z})_+$, where $\hat{z}$ denotes the maximum number of plasmids. Note that we will introduce below horizontal plasmid transfer, such that the number of plasmids may exceed the plasmid carrying capacity $\hat{z}$. In this case, we take the reproduction rate for plasmids $bz(1 - z/\hat{z})_+$ to zero (indicated by the index "+"). If we only consider the number of plasmids, the dynamics of $u_z(t)$ is described by the master equations for this birth-death process,

$$\dot{u}_z = -\,bz(1 - z/\hat{z})_+\ u_z + b(z-1)(1-(z-1)/\hat{z})_+\ u_{z-1}$$

where we formally define $u_{-1}(t) = 0$.

Now we consider a second effect: plasmid re-distribution via horizontal transmission. This is a kind of infection process: infected cells (cells with $z > 0$) infect other cells, undiscriminating cells without plasmids ($z = 0$) and cells with plasmids ($z > 0$). There are two fundamentally different approaches to model infection: mass action and standard incidence [4, 5]. We use here the standard incidence. At rate d a plasmid is transferred from a cell to a randomly selected cell within the population. We define the total amount of plasmid bearing cells

$$Z(t) = \sum_{z\in\mathbb{N}} u_z(t).$$

The parameter d denotes the rate at which a plasmids is lost (because it is transferred). Let furthermore $N(t) = \sum_{z'\in\mathbb{N}_0} u_{z'}$ denote the total population size. The probability that a given cell transfers the plasmid to a cell with z plasmids reads $u_z(t)/N(t)$. Hence, the rate at which a plasmid bearing cell transfers a plasmid is d, and the rate at which a cell receives a plasmid is $d\,Z/N$. We introduce the indicator function, $\chi_{>0}(z) = 1$ if $z > 0$ and $\chi_{>0}(z) = 0$ else. Then,

$$\begin{aligned}\dot{u}_z \quad = \quad & -[bz(1 - z/\hat{z})_+ + \chi_{z>0}(z)\,d + d\,Z(t)\,/N(t)]\ u_z + \chi_{z>0}(z+1)\,d\ u_{z+1} \\ & +[d\,Z(t)\,/N(t) + b(z-1)(1-(z-1)/\hat{z})_+]\ u_{z-1}.\end{aligned}$$

The following proposition indicates the consistency of the model in simple cases.

Prop.: *In the model developed so far, we always have $N'(t) = 0$. That is, the total population of cells is constant. Moreover, if $b = 0$, then $\frac{d}{dt}\sum_{z=\in\mathbb{N}} z\,u_z(t) = 0$ which does mean that the total number of plasmids is constant.*

Proof: First of all, we have

$$\begin{aligned} N' &= -\sum_{z=0}^{\infty}\left([bz(1-z/\hat{z})_+ + \chi_{z>0}(z)\,d + d\,Z(t)\,/N(t)]\;u_z\right) + \sum_{z=0}^{\infty}\left(\chi_{z>0}(z+1)\,d\;u_{z+1}\right) \\ &\quad + \sum_{z=0}^{\infty}\left([d\,Z(t)\,/N(t) + b(z-1)(1-(z-1)/\hat{z})_+]\;u_{z-1}\right) \\ &= -(dZ + d(Z/N)\,N) + d\,Z + (d\,Z/N)\,N = 0. \end{aligned}$$

Next we take $b = 0$, and find

$$\dot{u}_z = -[\chi_{z>0}(z)\,d + d\,Z(t)\,/N(t)]\;u_z + \chi_{z>0}(z+1)\,d\;u_{z+1} + d\,(Z(t)\,/N(t))\;u_{z-1}.$$

Hence,

$$\begin{aligned} \frac{d}{dt}\sum_{z=0}^{\infty} zu_z &= -\left(\sum_{z=0}^{\infty}[d\chi_{>0}(z) + d\,Z(t)\,/N(t)]\;z\,u_z\right) \\ &\quad + \left(\sum_{z=0}^{\infty} d\chi_{>0}(z+1)\,z\,u_{z+1}\right) + \left(\sum_{z=1}^{\infty}[d\,Z(t)\,/N(t)]\;z\,u_{z-1}\right) \\ &= -d\left(\sum_{z=0}^{\infty} zu_z\right) - d\,(Z(t)\,/N(t))\left(\sum_{z=0}^{\infty} zu_z\right) + d\left(\sum_{z=1}^{\infty}(z-1)\,u_z\right) \\ &\quad + d\,(Z(t)\,/N(t))\left(\sum_{z=0}^{\infty}(z+1)\,u_z\right) \\ &= 0 \end{aligned}$$

□

Dynamical processes II+III: Cell divisions and cell death. Cell division and cell death is added in the same way as we did in the main text. All in all, we obtain

$$\begin{aligned} \dot{u}_z &= -[bz(1-z/\hat{z})_+ + d\,\chi_{z>0}(z) + d\,Z(t)\,/N(t)]\;u_z \\ &\quad + d\,\chi_{z>0}(z+1)\;u_{z+1} + [d\,Z(t)\,/N(t) + b(z-1)(1-(z-1)/\hat{z})]\;u_{z-1} \\ &\quad -\beta(z,A)u_z + \sum_{z_0=z}^{\infty}[g(z;z_0) + g(z_0-z;z_0)]\beta(z_0,A)u_{z_0} - \mu(z,A)u_z. \end{aligned} \quad (1)$$

In contrast to the model in the main text, we have a nonlinear ODE. The right hand side of this ODE is homogeneous of degree one: if we define $\vec{u} = (u_0, u_1, \ldots)$, we may write the ordinary differential equation as $\vec{u}' = F(\vec{u}, A)$, where $F(\theta\vec{u}, A) = \theta F(\vec{u}, A)$ for $\theta > 0$ and $\vec{u} > 0$ (component wise).

Long term behavior. It is well known that population growth and distribution of the population structure can be separated in a system that is homogeneous of degree on [5]: Define $N(t) = \sum_{z\in\mathbb{N}_0} u_z(t)$, and $v(t) = u(t)/N(t)$.

Then,

$$N' = \left(\sum_{z \in \mathbb{N}_0} F_z(v, A) \right) N$$

$$v' = F(v, A) - \left(\sum_{z \in \mathbb{N}_0} F_z(v, A) \right) v.$$

Numerical simulations show that $v(t)$ becomes stationary in the long run. If v^* is the stationary state, then $F(v^*, A)$ indicates the long term growth rate of the population. As before, an ESSS is defines as the plasmid segregation strategy that maximizes that growth rate.

Parameters. The parameter d represents the rate at which plasmids are horizontally transferred. We use here standard incidence. The papers that estimated this parameter implicitly assumed mass action. In order to determine dm we need to multiply the rates estimated by experiments with a typical population size (density), which is typically in the range of 10^6 cells per ml. As the (mass action) transition rate is in the range of $10^{-10}/(h \text{ cell})$ [1, 2, 3], we choose

$$d = 0.0001/h$$

As above, we use $\beta_0 = 1/h$, $b = 1.2/h$ and $\hat{z} = 50$. In the present model, we need to specify explicitly the growth and eath rate for unprotected cells. We assume that cells without pasmids do not grow in the presence of antibiotics, and that they die at rate $5/h$.

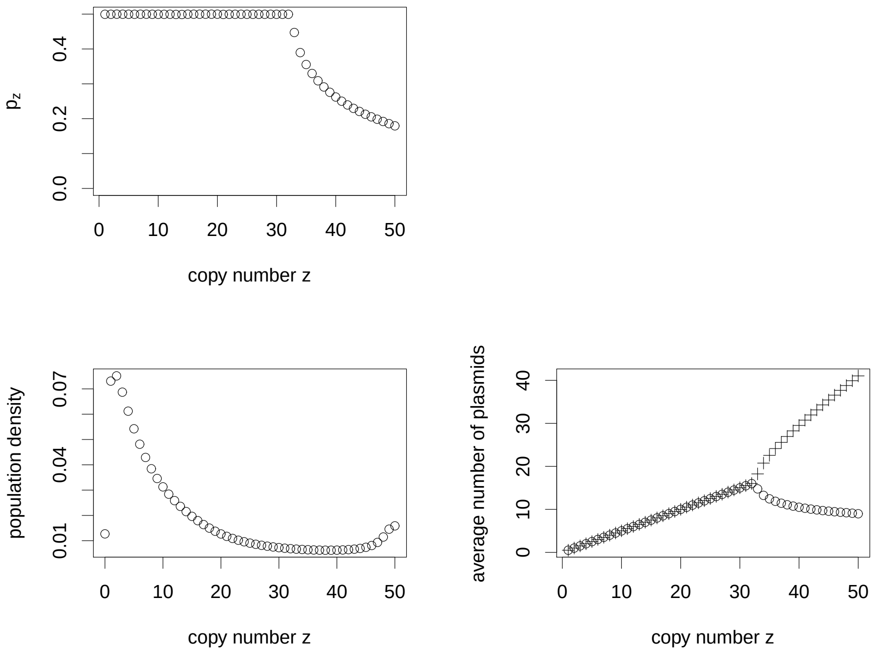

Figure K: $\beta_0 = 1/h$, $b = 1/h$, $\hat{z} = 50$, birth rate for unprotected cells is 0, death rate of unprotected cells is $5/h$, $d = 0.0001/h$. Note that the population density is not only shown for the plasmid-bearing cells, but also for $z = 0$.

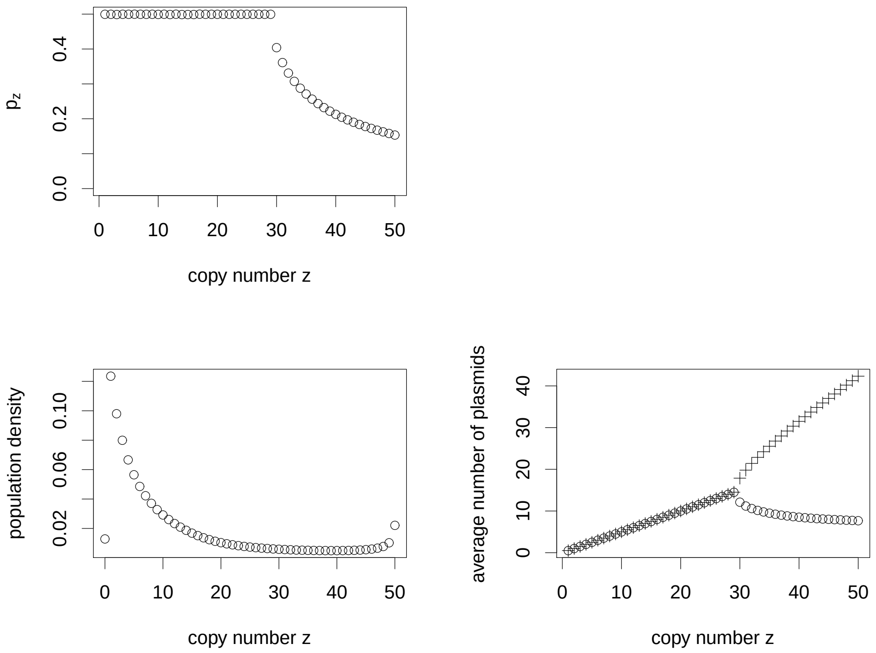

Figure L: $\beta_0 = 1/h$, $b = 1/h$, $\hat{z} = 50$, birth rate for unprotected cells is 0, death rate of unprotected cells is $5/h$, $d = 1/h$. Note that the population density is not only shown for the plasmid-bearing cells, but also for $z = 0$.

Numerical procedure. In order to estimate the asymptotic growth rate in an environment with antibiotics, we used an explicit Euler method with step width $h = 0.001/h$ and solve the ODE for $15h$, such that v approaches its stationary state. We average the growth rate for the next $4h$. To find the ESSS, we use the steepest ascent method as described in the main part of the paper.

Result. Horizontal plasmid transfer hardly makes an effect (Fig. 11, 12), even if we take the rate $d = 1/h$, which is approximately 1000 times of the parameters that adequately describes the biological system. If u_0 is small, $Z(t) \approx N(t)$. The rate to lose and receive a plasmid is about the same. That is, plasmid re-distribution will not change the invariant copy number distribution. Only if the plasmid-free population becomes large, there is a net flow of plasmids from cells with plasmids to plasmid-free cells, i.e. (as the plasmid-free cells receive a plasmid) to u_1.
If the rate to transmit a plasmid would heavily depend on the copy number (e.g. is linearly increasing with the copy number), then horizontal plasmid transfer would effectively re-shuffle plasmids in the population. In this case, we would find a distinct effect.

Monotonicity of the Fitness in a

We show that the fitness decreases in a in the F1 generation given a heuristic weight to the fitness that represents plasmid loss. Common positive constants are eliminated in the subsequent steps - only the sign of the derivative is of interest.

$$
\begin{aligned}
&\frac{d}{da}\left(q((1+a)z_1/2)\left(1-\frac{1}{2}(1+a)\,z_1/\hat{z}\right)+q((1-a)z_1/2)\left(1-\frac{1}{2}(1-a)\,z_1/\hat{z}\right)\right)\\
=\;& q'((1+a)z_1/2)\frac{z_1}{2}\left(1-\frac{1}{2}(1+a)\,z_1/\hat{z}\right)-q((1+a)z_1/2)\,\frac{1}{2\hat{z}}\\
&-q'((1-a)z_1/2)\frac{z_1}{2}\left(1-\frac{1}{2}(1-a)\,z_1/\hat{z}\right)+q((1-a)z_1/2)\frac{1}{2\hat{z}}\\
&\text{use: } q((1+a)z_1/2)>q((1-a)z_1/2),\quad q'((1+a)z_1/2)<q'((1-a)z_1/2)\quad \text{and } a>0\\
<\;& q((1+a)z_1/2)\frac{z_1}{2}\left\{\left(1-\frac{1}{2}(1+a)\,z_1/\hat{z}\right)-\left(1-\frac{1}{2}(1-a)\,z_1/\hat{z}\right)\right\}\\
<\;& q((1+a)z_1/2)\frac{z_1}{2}\left\{\left(\frac{1}{2}(-1-a)\,z_1/\hat{z}\right)+\left(\frac{1}{2}(1-a)\,z_1/\hat{z}\right)\right\}<0
\end{aligned}
$$

References

1. Levin BR, Stewart FM, Rice VA. The kinetics of conjugative plasmid transmission: fit of a simple mass action model. Plasmid. 1979;2:247–260.

2. Elsas JDV, Govaert JM, Veen JAV. Transfer of plasmid pFT30 between bacilli in soil as influenced by bacterial population dynamics and soil conditions. Soil Biology and Biochemistry. 1987;19(5):639–647. doi:10.1016/0038-0717(87)90110-6.

3. Lopatkin AJ, Meredith HR, Srimani JK, Pfeiffer C, Durrett R, You L. Persistence and reversal of plasmid-mediated antibiotic resistance. Nature communications. 2017;8:1689. doi:10.1038/s41467-017-01532-1.

4. Brauer F, van den Driessche P, Wu J, editors. Mathematical Epidemiology. Springer Berlin Heidelberg; 2008. Available from: http://www.ebook.de/de/product/25103478/mathematical_epidemiology.html.

5. Müller J, Kuttler C. Methods and Models in Mathematical Biology. Springer Berlin Heidelberg; 2015. Available from: http://dx.doi.org/10.1007/978-3-642-27251-6.
